# What Coronavirus Disease 2019 Has Taught Us About Modern Electrophysiology Practice

**DOI:** 10.19102/icrm.2020.110801

**Published:** 2020-08-15

**Authors:** Daniel Sohinki, Adam E. Berman

**Affiliations:** ^1^Division of Adult Cardiology, Medical College of Georgia, Augusta, GA, USA; ^2^Division of Pediatric Cardiology, Medical College of Georgia, Augusta, GA, USA

**Keywords:** COVID-19, remote monitoring, telemedicine

The impact of coronavirus disease 2019 (COVID-19) upon the global practice of medicine is seismic. Physicians and hospitals worldwide are facing unprecedented challenges, struggling to find a balance between appropriate care delivery, patient and staff safety, and dire economic conditions that threaten the livelihoods not only of patients but also of health care providers and the entire health system. Such existential threats demand innovations in health-care delivery and a re-examination of contemporary medical practice. This is especially relevant in subspecialty care, which serves as a microcosm of the public health response to COVID-19. As appropriate public health measures vary from state to state (eg, based on population density, geography, urbanization) so too do effective health-care delivery models between specialties.

Cardiac electrophysiology (EP) has been uniquely impacted by the COVID-19 pandemic. Being the prototype “elective procedure” specialty, EP physicians have been redeployed, forced to take on an expanded consultative role or to reduce the amount of time they work. As such, clinical and procedural EP volumes have dropped precipitously.

## Toward an augmented electrophysiology practice: the role of telehealth

**Figure 1:**
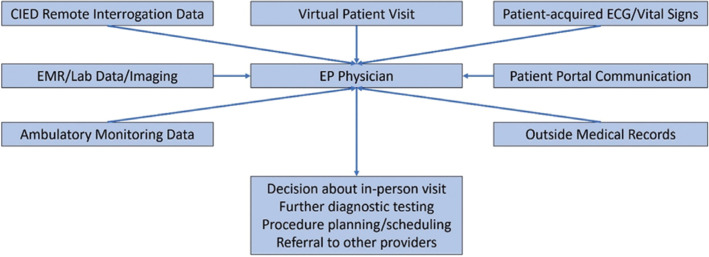
A theoretical framework for a virtual EP practice. The EP physician has access to a wide variety of data in addition to history information gathered from the virtual visit with the patient. It is then up to the physician’s discretion to decide whether a formal in-person visit is necessary or if appropriate care can be coordinated using available data. EMR: electronic medical record; EP: electrophysiology.

In response to these challenges, many within the EP community have reexamined their practices, analyzing what is actually required to deliver effective care. One revelation during the COVID-19 pandemic has been the robust access to care that can be provided by telemedicine services. EP seems uniquely suited to adopt telemedicine as a care delivery platform as an undeniably technical specialty that is increasingly reliant on objective, pre-acquired data in a manner that does not require the patient to physically interact with a physician to share in clinical decision-making **(Figure 1)**. Obtaining a patient’s subjective history is, in many ways, augmented via telemedicine’s heightened focus on interpersonal communication.

When surveyed, patients largely support virtual encounters. A 2017 survey conducted by The Advisory Board Company™ (Washington, D.C., USA) revealed that 77% of surveyed patients would consider seeing a physician virtually and that nearly 20% already had.^1^ Telemedicine has previously found support in other specialties, with a majority of patients willing to engage in virtual visits in the area of pediatrics as well as other chronic conditions.^2^ Across studies, patients cite the ability to stay home, not having to sit in a waiting room, and a lack of required travel as reasons for why they view telemedicine favorably. In fact, a majority of patients express a willingness to use telehealth for preprocedure, selected postprocedure, and chronic disease management visits.^1^ A large study using Consumer Assessment of Healthcare Providers and Systems data demonstrated no difference in patient satisfaction when comparing in-person and telehealth visits.^3^

These findings lead to a crucial question—does a plurality of EP patients actually need to see an electrophysiologist in person to receive appropriate care? With minimal instruction, patients could be taught to record their own blood pressure, heart rate, and temperature prior to dialoguing with their physician via telehealth. Likewise, the EP physician can review the patient’s chart electronically and document in real time. Patients could be referred for laboratory or imaging testing that could be completed locally, which is particularly advantageous for patients who live long distances from their specialist. These results can then be communicated to the physician via electronic health records, fax, secure messaging, or the telemedicine platform itself, thereby ensuring longitudinal care.

Prior challenges in the acquisition of ambulatory electrocardiography (ECG) would have presented a significant impediment to the implementation of a virtual EP practice. Today, however, with the advent of smartphone and smartwatch technologies, consumer electronics have become an attractive modality for ambulatory ECG recording, capable of rapid, high-quality rhythm strip acquisition and transmission, enabling remote physician review. The KardiaMobile™ smartphone monitor (AliveCor, Mountain View, CA, USA) enables patients to record a single channel or six-lead ECG using a handheld electrode pair that interfaces with their smartphone or tablet, which has been shown to demonstrate high sensitivity and specificity for atrial fibrillation (AF) detection^4^ and similar diagnostic yield as a 14-day ambulatory monitor.^5^ Similarly, the QardioCore™ ECG monitor (Qardio, Inc., San Francisco, CA, USA) is a wearable device capable of recording up to three ECG channels that pairs with the patient’s smartphone or tablet. Data from these devices may be sent directly to the physician or may be uploaded and viewed on secure patient portals. Ambulatory blood pressure monitoring solutions are available from both companies, allowing patients a centralized way to track and transmit their hemodynamics to their physician. Even the Apple Watch™ (Apple, Cupertino, CA, USA) is capable of recording and transmitting a single-lead ECG, with an AF detection algorithm found to exhibit a relatively high positive predictive value.^6^ **Table 1** lists commonly used consumer electronic solutions adopted for arrhythmia monitoring and their associated costs, efficacy, and patient compliance rates as derived from published studies.

Cardiac implantable electronic devices (CIEDs) represent another vital source of remote data. These devices are capable of storing detailed information on arrhythmia episodes, premature ventricular contraction and AF burden, daily heart rate range, and device-delivered therapies. Advances in accelerometer and thoracic impedance measurement technology also enable the monitoring of patient activity and clinical volume status. These data can be transmitted remotely to the physician via manufacturer-specific networks facilitating care planning while empowering the physician to make real-time therapeutic adjustments that may prevent unnecessary office visits and hospital admissions.^7^ The remote monitoring of CIEDs has also been shown to reduce health-care utilization, decrease time to clinical decision-making, and reduce inappropriate shocks in patients with implantable cardioverter-defibrillators.^8^

We acknowledge that telemedicine is not appropriate for all patients. Certain degrees of technical savviness and health literacy are required on the patient’s part to enable them to participate fully in the visit. A small pilot study explored the feasibility of nurse-supported telemedicine visits among seniors with low levels of computer literacy, with visits lasting 20 to 25 minutes. Notably, a majority of participants (7/10) felt they needed additional instruction to effectively utilize the telehealth platform.^9^ A current limitation of telemedicine is the requirement that the patient possesses a device capable of connecting to their provider’s telemedicine platform and the ability to navigate the platform to “arrive” for the visit. Patients should also be able to measure their own blood pressure, heart rate, and temperature using commercially available products. These perceived encumbrances, however, may serve to nudge patients to further invest in their own health and should not be viewed as impediments to the implementation of telemedicine. We acknowledge that the impact of converting a significant number of clinic visits to telehealth visits on clinic efficiency and overall practice economics is currently unknown and deserves further investigation.

## Challenges of telehealth—perceived and real

Despite these advantages, telemedicine has been criticized for removing crucial physical contact between the patient and physician. How does one perform a complete assessment of the patient without a physical examination? In response to these concerns, let us acknowledge some basic realities. First, it would be impractical to practice telemedicine exclusively. As with other aspects of medicine, a periodic comprehensive evaluation including a physical examination is crucial in facilitating patient selection for invasive procedures. It is our experience that most referring physicians expect to conduct an in-person consultation during new patient visits. Despite these conventions, telehealth can play a role for new patient evaluations in select circumstances. Having been “screened” via telehealth, a new patient with a particular physical complaint that requires direct examination can always follow up with the physician in person for a complete examination. Although these concerns are real, a plurality of EP patients may exist for whom an evidence-based diagnostic and therapeutic plan may be effectively implemented using telehealth. Accordingly, we recall one of Osler’s most important admonitions: “listen to your patient because they are telling you their diagnosis.”^10^

Payers have also recognized that physician reimbursement should be updated to allow telemedicine to be sustainable. On April 1, 2020, the Centers for Medicare and Medicaid Services announced revisions to fee schedules for telehealth evaluation and management (E/M) codes. Under the new guidelines, reimbursement is exactly the same for in-person encounters for visit levels 1 through 4.^11^ Codes also exist for telephone-only encounters, which are now reimbursed at rates similar to in-person visits.^11^ Medicare guidelines have been updated to allow “distant sites” (eg, rural health clinics) to bill for telehealth services, affording critical access for patients in rural and underserved areas.^11^ Physician review of patient-submitted images and telephone communication is also billable, ensuring reimbursement is gained for these virtual services. The COVID-19 pandemic has also accelerated widespread adoption among payers in the United States to appropriately reimburse providers participating in telehealth. We anticipate that these changes will be continued following resolution of the current crisis, recognizing that access to care is vital to our patients’ and nation’s health. Historical discounting by insurers of cardiovascular care delivered by telehealth means should be reconsidered in light of the effectiveness of telehealth-care delivery during this unprecedented pandemic. Further cost-effectiveness analysis of the impact of telehealth in EP care delivery appears to be a robust area of future investigation, as data suggesting the economic impact of converting a significant proportion of a practice to a telehealth platform are currently lacking.

## The “tele-electrophysiology” practice of tomorrow

Technological innovation is to be embraced in the modern practice of medicine, especially when it improves access to care. Extrapolating beyond the traditional E/M encounter to our procedural practice, we consider another iconoclastic concept—does the operating physician need to actually be at the bedside to perform certain invasive procedures? Advances in remote magnetic navigation technology have enabled precise intracardiac catheter manipulation to be conducted from a remote desktop keyboard. Via enhanced interhospital communication technology, the ability to consult with a remote first-assisting “telesurgeon” during a live procedure potentially represents a new frontier in expanding access to highly specialized expert care outside of major medical centers.

Viewing telehealth within the ecosystem of EP practice, we should be open to acknowledging that some sacrosanct aspects of medicine may in fact be an impediment to a patient’s access to care—and are areas where advances in our understanding of technology’s role may expand our capacity to provide exceptional patient care. While the COVID-19 pandemic has engendered unprecedented human suffering at a global scale, our response to the challenges it has posed to the practice of EP should include embracing and actuating the technologies that help preserve the sacred doctor–patient relationship while enhancing patient care.

## Figures and Tables

**Table 1:** Clinical Performance and Cost of Consumer-based Arrhythmia Monitoring Solutions

	AF Detection	Any Arrhythmia Diagnosis	Patient Compliance	Cost
KardiaMobile™ (AliveCor, Mountain View, CA, USA)	Sensitivity: 87.0%, specificity: 97.9%^5^	90-day diagnostic yield: 55.6%^12^	43.2% of participants successfully transmitted^12^	$89.00–149.00
Apple Watch™ (Apple, Cupertino, CA, USA)	Positive predictive value: 71%^6^	Not studied	76% of notified patients followed up with^6^	$399.00
QardioCore™ (Qardio, Inc., San Francisco, CA, USA)	Not FDA-approved for AF detection	Not studied	Not studied	£359.00*
EKGraph (SonoHealth, Charleston, SC, USA)	Not FDA-approved for AF detection	Not studied	Not studied	$119.00
Hexoskin™ (Carré Technologies Inc., Montreal, QC, Canada)	Not FDA-approved for AF detection	Not studied	Not studied	$249.00–579.00

AF: atrial fibrillation; FDA: Food and Drug Administration.

As noted, the two most studied products are the KardiaMobile™ device and the Apple Watch™.

*Not currently for sale in the United States.
